# Aggressive Postoperative Rehabilitation With Platelet-Rich Plasma Therapy for a Lauge-Hansen Supination-External Rotation Type IV Ankle Fracture: A Case Report of Early Return to Sports

**DOI:** 10.7759/cureus.77673

**Published:** 2025-01-19

**Authors:** Hiroyuki Omiya, Minami Kawamura, Shinnosuke Hada

**Affiliations:** 1 Department of Rehabilitation, Tokyo Rosai Hospital, Tokyo, JPN; 2 Department of Orthopaedics, Juntendo University, Tokyo, JPN; 3 Department of Orthopaedics, Hada Medical Clinic, Tokyo, JPN

**Keywords:** accelerated return to sport, aggressive postoperative rehabilitation, early weight-bearing, platelet-rich plasma, ser type stage iv fracture

## Abstract

Supination-external rotation (SER) type IV ankle fractures, as classified by Lauge-Hansen (L-H), are highly unstable due to frequent injuries of the deltoid and distal tibiofibular ligaments. These fractures typically require open reduction and internal fixation (ORIF), followed by prolonged immobilization and delayed weight bearing. Such protocols often result in extended recovery periods, delaying the return to competitive sports. In the clinical setting, there is a growing demand for approaches that enable athletes to resume sports quickly. Platelet-rich plasma (PRP) therapy has demonstrated the potential to enhance bone and soft tissue healing. Although PRP is widely used in ligament and tendon injuries, PRP’s role in fracture rehabilitation is less well established, and it is often considered supplementary. We report the case of a 17-year-old male high school rugby player who sustained an SER type stage IV ankle fracture during a match. ORIF was performed eight days post-injury using a 1/3 tubular plate and the Ziptight system. Postoperatively, the patient underwent two weeks of cast immobilization and non-weight-bearing rest. After two weeks (postoperative day 15), the cast was removed. Radiographic and ultrasonographic evaluations confirmed the fracture stability without displacement and early callus formation. Based on these findings, full weight-bearing ambulation was permitted under close supervision, as part of a structured rehabilitation plan. By the fifth week, the patient achieved full range of motion (ROM), allowing him to begin jogging. Temporary tendon gliding issues in the seventh week were treated with hydrodissection, which enabled the resumption of partial training. By the eighth week, the patient was cleared for competitive rugby. At the one-year follow-up, he reported no pain, deformity, or functional limitations and had fully resumed his preinjury level of play. This case report highlights the potential of aggressive rehabilitation combined with PRP therapy to expedite recovery in SER type stage IV ankle fractures. Further research is needed to validate these findings in larger cohorts.

## Introduction

The Lauge-Hansen (L-H) classification of supination-external rotation (SER) type stage IV ankle fractures is characterized by significant instability due to the frequent involvement of deltoid ligament injuries and distal tibiofibular ligament damage. Open reduction and internal fixation (ORIF) is the recommended standard treatment, typically followed by prolonged weight-bearing restrictions and cast immobilization [1].

Standard rehabilitation protocols typically include two weeks of cast immobilization, weight-bearing initiation at six weeks, and functional recovery programs after 10 weeks [2,3]. However, these protocols often result in prolonged recovery periods, delaying the return to competitive sports. Prolonged immobilization and delayed weight-bearing can cause joint contractures, muscle atrophy, and hindered recovery, emphasizing the importance of early weight-bearing [4]. From the perspective of accelerating postoperative rehabilitation, several studies have demonstrated the potential benefits of autologous biotherapy in enhancing the healing process for various musculoskeletal injuries. Specifically, platelet-rich plasma (PRP), created by centrifuging a sample of a patient’s blood to isolate the platelets, has been gaining attention [5]. The treatment depends on the ability of growth factors and anti-inflammatory cytokines contained in platelets to promote the repair of damaged tissues.

PRP delivers growth factors such as platelet-derived growth factor (PDGF), transforming growth factor-beta (TGF-β), and vascular endothelial growth factor (VEGF), which collectively contribute to promoting cell proliferation and improving tissue regeneration [6,7]. Treatments utilizing platelets, including PRP, have proven effective in promoting fracture healing by activating mesenchymal progenitor cells and osteoblasts, with a randomized controlled trial reporting an 87.5% union rate using platelet-rich fibrin compared to 50% in the control group for open tibial fractures [6,7].

While PRP therapy has been increasingly used for muscle, tendon, and ligament injuries to support an early return to sports, its application in fracture rehabilitation, particularly in aggressive protocols for SER type stage IV ankle fractures, remains underexplored. Here, we report the case of a high school rugby player with an SER type stage IV ankle fracture who underwent aggressive rehabilitation combined with PRP therapy, aiming to contribute to the understanding of its potential for expedited recovery and early return to sports.

Written informed consent for academic use and publication of this data was obtained from the patient and is available upon request.

## Case presentation

**Figure 1:**
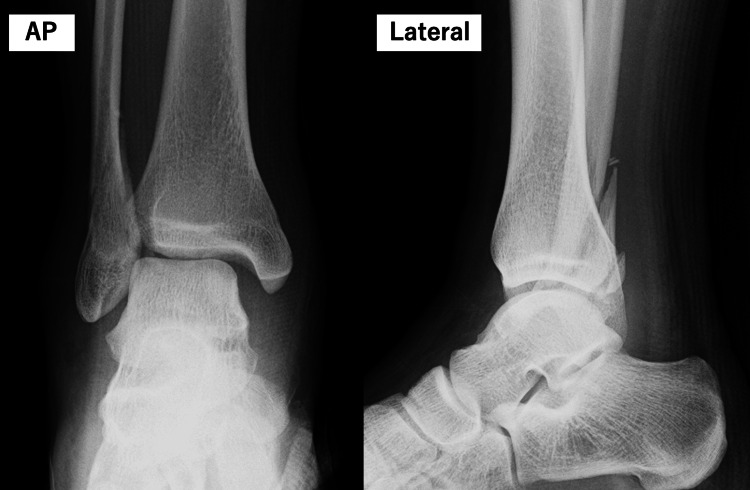
Preoperative X-rays of the right ankle Preoperative anteroposterior and lateral X-ray views of the right ankle demonstrating a SER type stage IV fracture as classified by L-H. L-H, Lauge-Hansen; SER, supination-external rotation

**Figure 2:**
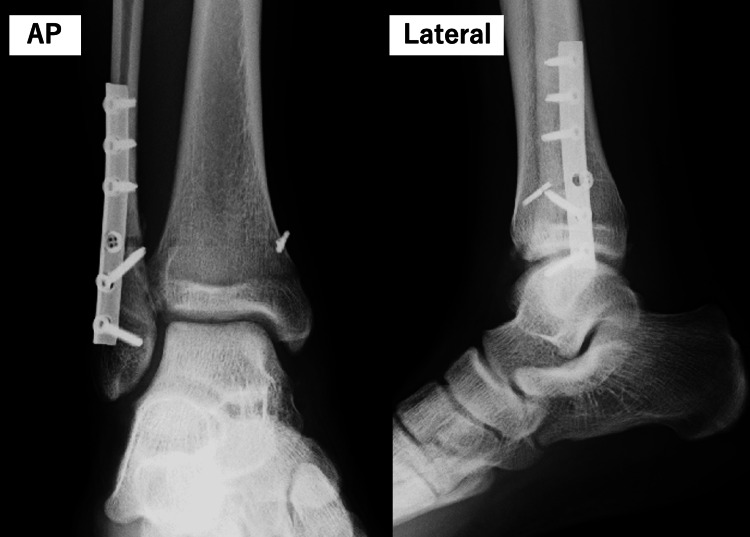
Postoperative X-rays of the right ankle Postoperative anteroposterior and lateral X-ray views of the right ankle following ORIF. The fixation was performed using a 1/3 tubular plate and Ziptight system. ORIF, open reduction and internal fixation

A 17-year-old male high school rugby player sustained a right ankle injury when tackled during a match while his foot was fixed. Radiographs obtained, showing the anteroposterior and lateral views on the day of injury, confirmed an L-H SER type IV ankle fracture (Figure 1). Eight days post-injury, ORIF was performed using a 1/3 tubular plate and the Ziptight system (Zimmer Biomet Holdings, Inc., Warsaw, Indiana, USA) (Figure 2). The patient and his family expressed a strong desire for an early return to rugby within eight weeks of surgery. After understanding the benefits and risks, they agreed to an aggressive rehabilitation protocol incorporating PRP therapy to support early recovery and weight-bearing.

Postoperatively, the patient underwent two weeks of cast immobilization and non-weight bearing. After two weeks (postoperative day 15), the cast was removed. PRP therapy was administered using the GPS III system (Zimmer Biomet Holdings, Inc., USA). The PRP was injected into both the fracture site and the surrounding injured ligaments, including the deltoid ligament and distal tibiofibular ligament, under ultrasound guidance to ensure precise delivery. Radiographic and ultrasonographic evaluations confirmed fracture stability without displacement and early callus formation. Based on these findings and the initiation of PRP therapy, full weight-bearing ambulation was permitted under close supervision, as part of a structured rehabilitation plan. The rehabilitation plan (Table 1) was designed to achieve the goal of returning to competitive rugby by the eighth postoperative week. At the time of cast removal, the range of motion (ROM) was 0° dorsiflexion and 30° plantar flexion, with pain localized below the lateral malleolus under partial weight-bearing.

**Table 1:** Postoperative rehabilitation and orthopedic management plan US: ultrasonography, PRP: platelet-rich plasma, ROM: range of motion, DUTY: duty cycle (in ultrasound therapy), CT: computed tomography, EHL: extensor hallucis longus

Postoperative week	Orthopedic assessment and procedures	Instructions from orthopedist	PT training (only changes highlighted)
Week 1-2 (days 0-14)	X-ray	Non-weight-bearing	PT not yet initiated
	Cast immobilization		
Week 3 (days 15-21)	X-ray/US	Full weight-bearing	Ultrasound therapy (3MHz, DUTY 50%)
	Cast removal		Soft tissue mobilization
	PRP therapy administered to the fracture site and ligaments		Active-passive ROM ex.
		ROM: free	Towel gathering exercise
			Seated calf raises
Week 4 (days 22-28)	X-ray/US	Resisted ankle joint training	Ultrasound therapy (3MHz, DUTY 100%)
			Isometric inversion/eversion ankle training
			Standing calf raises
			Mini-squats
Week 5 (days 29-35)	X-ray/US/CT	Jogging and light agility training	Single-leg calf raises (isometric contraction)
			Single-leg squats
			Jogging
Week 6 (days 36-42)	X-ray/US	Sprinting	Single-leg calf raises (concentric and eccentric contraction)
	Hydrodissection for EHL tendon		Light agility training
Week 7 (days 43-49)	No orthopedic consultation	Partial return to sport permitted	Single-leg single jumps
Week 8 (days 50-56)	X-ray/US	Full return to sport permitted	Single-leg continuous jumps
	Hydrodissection for EHL tendon		Agility training

The rehabilitation focused on enhancing soft tissue flexibility using ultrasound therapy and progressively strengthening the peri-ankle muscles. Ultrasound therapy was performed using the EU-910 device (Ito Co., Ltd., Japan). In the third postoperative week, ultrasound therapy aimed to reduce inflammation and promote early soft tissue repair through the thermal and non-thermal effects of ultrasound. This was conducted at a frequency of 3 MHz, a duty cycle of 50%, and an intensity of 1.0 W/cm². In the fourth week, therapy settings were adjusted to a 100% duty cycle to achieve an approximate 4°C increase in the temperature of the soft tissues, enhancing flexibility. Concurrently, soft tissue mobilization was performed to further improve flexibility and reduce adhesions. By the fifth postoperative week, full ROM was restored, and jogging was commenced upon achieving unilateral calf raises. 

In the sixth postoperative week, restricted gliding movement of the extensor hallucis longus (EHL) tendon caused pain. To address this issue, a hydrodissection was performed by an orthopedic surgeon under ultrasound guidance. The surgeon evaluated the EHL tendon movement and identified specific areas of adhesion with surrounding tissues. A solution consisting of 1% lidocaine (3 mL) and 5% dextrose (7 mL) was injected into the adhesion sites. This procedure effectively released the adhesions, alleviated pain, and enabled a progressive increase in the intensity of rehabilitation.

By the eighth week, the patient was cleared for competitive rugby. At the one-year follow-up, the patient reported no pain, deformity, or functional limitations and participated in rugby at the pre-injury level without complications.

## Discussion

Several factors contributed to the patient’s successful early return to competitive sports. The first was the implementation of aggressive rehabilitation through early postoperative weight-bearing. Porter et al. outlined a phased recovery program for distal tibiofibular ligament injuries: phase I (weeks 1-4) focuses on pain reduction and ROM improvement, phase II (weeks 4-8) emphasizes further ROM and strength recovery, and phase III (week 8 onward) targets functional activities [3]. In the present case, a condensed version of these phases facilitated early functional recovery with minimal complications. Although early weight-bearing is often avoided due to concerns about risks such as fragment displacement and ligament reinjury, studies have shown that it does not adversely affect surgical outcomes and may even accelerate functional recovery. Controlled loading of soft tissues, tailored to tissue conditions, optimizes recovery by enhancing metabolic activity, blood flow, and nutrient delivery, which supports cellular regeneration and collagen remodeling [8]. For example, early weight-bearing groups have demonstrated superior dorsiflexion (15° vs. 10°) and plantarflexion (35° vs. 25°) at six weeks, faster walking capacity (Timed Up and Go test: 12 s vs. 15 s), and reduced swelling compared with delayed weight-bearing groups [4].

Traditionally, radiographic detection of callus has been the standard for determining the progression of weight-bearing stages. However, in this case, ultrasonography, reported to detect callus formation 23% earlier than radiography, was also incorporated as an assessment tool, which facilitated the decision to initiate early weight-bearing [9].

The second factor was the use of PRP therapy. PRP therapy likely supported early inflammation resolution and tissue healing by delivering growth factors such as PDGF, TGF-β, and VEGF [6]. A previous study showed that PRP increased bone healing rates by 1.6-2.2 times in bone defects [10]. While PRP appears to be beneficial, its specific contribution is challenging to isolate owing to the multifactorial nature of rehabilitation. Further studies are required to validate their effectiveness in similar contexts.

This case suggests that combining aggressive postoperative rehabilitation with PRP therapy may facilitate an earlier return to sports for athletes with SER type stage IV ankle fractures. This approach has the potential to serve as an alternative to traditional rehabilitation protocols, which often result in delayed recovery. However, further studies involving larger patient populations and controlled trials are needed to confirm these findings.

## Conclusions

Aggressive rehabilitation combined with PRP therapy allowed the patient to return to competitive rugby eight weeks post-surgery for an L-H SER type IV ankle fracture, with no complications or issues arising during the recovery process. In conclusion, this case demonstrates that combining aggressive postoperative rehabilitation with PRP therapy can facilitate an earlier return to sports for athletes with SER type stage IV ankle fractures. This approach may offer a promising alternative to traditional rehabilitation protocols, which often result in delayed recovery. Further investigation involving larger patient populations and controlled trials is essential to confirm these findings.
